# Supplementary material for: Effects of Mediterranean Diet or Low-Fat Diet on Blood Fatty Acids in Patients with Coronary Heart Disease. A Randomized Intervention Study
Source: Nutrients. 2021 Jul 13;13(7):2389. doi: 10.3390/nu13072389 (PMC8308706; doi:10.3390/nu13072389)
Supplement: Supplementary file 1 [file nutrients-13-02389-s001.zip › nutrients-1282778-supplementary.pdf]

Table S1: Correlations between the blood fatty acids profile and each item of PREDIMED questionnaire after three months of nutritional intervention (n=120).

PREDIMED Items / Fatty acids	Olive oil per day	Vegetable servings per day	Fruit units per day	sweet or carbonated beverages per day	Wine per week	Servings of fish or shellfish per week	Servings of nuts per week
**Total Saturated Fatty Acids**					0.23 **(0.01)**		-0.23 **(0.01)**
**16:0 (Palmitic Acid)**					0.33 **(0.0002)**		-0.34 **(0.0002)**
**Total Monounsaturated Fatty Acids**							-0.29 **(0.001)**
**16:1 (Palmitoleic Acid)**				0.23 **(0.01)**			-0.26 **(0.004)**
**18:1 (Oleic Acid)**							-0.27 **(0.003)**
**24:1**	0.26 **(0.004)**	0.25 **(0.007)**					
**20:3 n-9 (Mead Acid)**			-0.23 **(0.01)**			-0.27 **(0.003)**	
**Total Polyunsaturated Fatty Acids**							0.37 **(<.0001)**
**Total n-6**					-0.24 **(0.01)**		0.33 **(0.0003)**
**18:2 n-6 (LA)**							0.31 **(0.001)**
**22:4n-6**		-0.27 **(0.004)**				-0.42 **(<.0001)**	
**22:5 n-6 (DPA n-6)**						-0.32 **(0.0005)**	
**Total n-3**						0.26 **(0.004)**	0.24 **(0.007)**
**18:3 n-3 (ALA)**							0.32 **(0.0004)**
**20: 5 n-3 (EPA)**						0.38 **(<.0001)**	0.31 **(0.001)**
**22:6 n-3 (DHA)**						0.25 **(0.006)**	
**EPA+DHA**						**0.29 (0.001)**	
**Total n-6/Total n-3**						-0.33 **(0.0002)**	

Spearman's correlation r and (p-value). Only food items and fatty acid species with significant correlations p<0.01 were included in the table.

LA: Linoleic Acid; GLA: γ-Linolenic Acid; DHGLA: diomo-γ-Linolenic Acid; AA: Arachidonic Acid; DPA: Docosapentaenoic Acid; ALA: α-Linolenic Acid; EPA: Eicosapentaenoic Acid; DHA: Docosahexaenoic Acid.
